# The Relationship Between Preschool Teachers’ Proactive Personality and Innovative Behavior: The Chain-Mediated Role of Error Management Climate and Self-Efficacy

**DOI:** 10.3389/fpsyg.2021.734484

**Published:** 2021-11-04

**Authors:** Baocheng Pan, Zhanmei Song, Youli Wang

**Affiliations:** School of Education, Wenzhou University, Wenzhou, China

**Keywords:** preschool teachers, proactive personality, innovation behavior, error management climate, self-efficacy

## Abstract

**Objective**: This study, aims to explore the relationship of error management climate and self-efficacy between preschool teachers’ proactive personality and innovative behavior.

**Methods**: Four hundred thirty-nine preschool teachers were tested by proactive personality scale, error management climate scale, general self-efficacy scale, and employee innovation behavior scale.

**Results**: Preschool teachers’ proactive personality can directly predict their innovative behaviors, has a significant indirect effect on innovative behaviors through error management climate, and has a significant indirect effect on innovative behaviors through self-efficacy. Error management climate and self-efficacy play a chain-mediated role in the relationship between preschool teachers’ proactive personality and innovative behavior.

**Conclusion**: Error management climate and self-efficacy play a chain-mediated role in the relationship between preschool teachers’ proactive personality and innovative behavior.

## Introduction

There is little doubt that innovation is vital for organizational success and competitive advantage as well as for strong education in the 21st century (Mumford and Gustafson, 1988). Hence, an increasing attention is paid to creativity and innovation in today’s world. More and more organizations are constantly seeking to maximize their capabilities to adjust to dynamic environments, and at the same time to generate innovation (Reuveni and Vashdi, 2015). There is a close relationship between innovation and creativity (Robinson and Beesley, 2010), in which creativity refers to the emergence of new ideas (Robinson and Beesley, 2010), and innovation requires the realization of these ideas. Therefore, in order to achieve good organizational performance, the organization must promote the creative behavior of employees as much as possible (Nieves et al., 2014).

All over the world, educational management departments and schools in different countries will carry out educational innovation and reform in different periods to achieve the purpose of improving the quality of teaching (Runhaar et al., 2013). As a result, schools at all levels continue to innovate in education. With the development of social division of labor and education as a professional activity, competition and complexity have become an important feature of the education system (Miller, 2002). Schools are faced with new challenges and tasks when entering a new stage of development, which emphasizes school reform and school innovation, which will become a collective effort (Bryk et al., 1999). The success of school reform depends fundamentally on whether teachers are willing to innovate and realize the goal of school reform and development. It also requires that during organizational change, when the definition of work is unclear, schools will have to rely more on teachers who are willing to contribute to successful change, regardless of formal job requirements. At the same time, due to the complexity of innovation, schools are required to adopt a more tolerant attitude toward teachers’ innovative behaviors (DiPaola and Hoy, 2005).

Individual innovation behavior refers to the behavior that individuals discover problems, generate innovative ideas or solutions, seek support for their innovative ideas, put them into practice, and finally form commercial products or services (Scott and Bruce, 1994). In the kindergarten organization, the innovative behavior of preschool teachers is finally reflected in the aspect of educational service activities including the innovation of teaching activities, curriculum, and game activity organization. Research shows that individual innovation behavior helps employees themselves, groups, and organizations to better complete tasks (Janssen, 2000).

At present, the research on the influencing factors of individual innovation behavior mainly focuses on the aspects of individual intelligence (Wu et al., 2014; Örnek and Ayas, 2015), task characteristics (Dorenbosch et al., 2005), work environment (Lee and Hong, 2014), and leadership behavior (De Jong and Den Hartog, 2007; Reuvers et al., 2008). However, the research on the organizational factors of individual innovation behavior, especially the mismanagement climate, and the relationship between initiative and individual self-efficacy and individual innovation behavior is very limited (Maurer et al., 2017). Therefore, to further clarify the generation mechanism of preschool teachers’ innovative behavior is of great significance for the improvement of preschool teachers’ innovative ability and organizational innovation level. This study explores the relationship between proactive personality, error management climate, self-efficacy, and innovative behavior through empirical research.

## Literature Review and Theoretical Hypotheses

### Proactive Personality and Innovative Behavior

Bateman and Crant (1993) put forward the concept of proactive personality for the first time when they discussed the active component of organizational behavior; that is, a relatively stable personality or behavioral tendency of individuals who take active behaviors to influence the surrounding environment. Research shows that individuals with proactive personality tend to actively improve the existing environment or create a new environment (Crant and Bateman, 2000) and take the initiative to challenge the status quo rather than passively adapt and then show active change behavior. In school education, teachers with active personality and positive psychological characteristics will take active behaviors to change the existing environment and form innovative behaviors (Li et al., 2017). The research also proves that teachers with high proactive personality are more willing to integrate into the environment of change and are more likely to show more innovative behaviors in teaching and research work (Li et al., 2017).

Creative behavior, which is the generation of new ideas and the behavior of translating those ideas into action (Mumford and Gustafson, 1988), has long been regarded as one of the most important ways to judge the success of a product at the highest level of science, market, technology, and education (Mumford et al., 2002b). The people who are considered to have the greatest impact on their field, and the world at large are also often considered to be the ones with the most creative and innovative behavior in their field (Mumford et al., 2002a). Many studies have explained that creative behavior is related to unique factors such as motivation and climate (e.g., Mumford et al., 2007; Hirst et al., 2009; Tierney and Farmer, 2011). Firstly, from an individual perspective this interest in the task itself, intrinsic motivation-leads to a deeper and more intensive engagement with the task, which usually results in creativity (Amabile, 1996). Secondly, creativity is often enacted in team settings (Taggar, 2002), and a given team context is likely to influence the extent to which individuals act according to their dispositions (Mischel, 1977; Kristof, 1996). Therefore, the atmosphere factors in the team, especially the encouraging environment for innovation and the tolerant atmosphere for errors, will have an impact on individual innovation behavior.

In school, teachers’ creative behavior mostly takes place in class. Teachers are creative role models in the classroom. Students learn from teachers’ creative personality and behavior (Cropley, 1994). Creative teachers are effective in cultivating students’ creative development (Fryer and Collings, 1991). A creative teacher must possess a general capacity for self-improvement; recognize the integrity of a student personality’s development, formation of personality, and the harmony of people as the focus of education; be ready for dialog and understanding the students, be able for improvisation, and collective and individual creativity; create and maintain the atmosphere of mutual respect, mutual tolerance, and openness to criticism and innovation (Gorshunova et al., 2014).

However, teachers’ creative behaviors should not only be included in teaching behaviors but also in other activities, especially in the organization of kindergarten, where games are children basic activities, so they should also be included in the creation of game environment, game support, game observation, and other aspects. Early childhood is a very important period, during this period, children should be in cognitive, language, health, psychological, social, and other aspects of the overall and high-quality support. Dhieni et al. (2019) argue that those working in the field of early childhood education should be proactive and creative in order to understand how to enable children with great developmental potential to use their potential at the highest level and to provide high-quality activities for children in this regard. Preschool teachers provide creative environment and good atmosphere for children; for example, in kindergarten, teachers should have a good view of children and games to create a good environment for children games. Play provides a flexible atmosphere and encourages creative thinking because children are playing and are actually doing problem solving tasks (Dansky, 1980; Vandenberg, 1980; Pepler and Ross, 1981). In the course of the game, children easily explore and experiment with various solutions to different problems. In the course of this game, children’s creativity is enhanced. In the organization, employee innovation behavior has always been regarded as an important factor that can promote organizational innovation and maintain the sustainable development of the organization (Vessey et al., 2014; Zhou and Hoever, 2014). In the organization itself, there is no stipulation that employees must show innovative behavior (Parker and Collins, 2010). Whether employees make innovative behaviors is not subject to external coercive constraints, and it is more dependent on the creative behavior tendency of employees, which is a kind of initiative behavior.

Proactive personality is a stable individual difference variable affecting active behavior, which can positively predict all proactive behaviors (Parker and Collins, 2010) including individual innovation behaviors. Studies have found that, in organizations, employees with high proactive personality usually have positive quality and higher value pursuit. They are good at finding problems, seeking and seizing opportunities, and taking active actions to promote organizational change, so as to show more innovative behaviors conducive to the development and improvement of the organization (Bateman and Crant, 1993). Therefore, in an organizational environment, initiative is critical for both the individual and the organization. Empirical studies also show that proactive personality has a positive effect on creativity such as generating innovative ideas or proposing solutions to problems (Kim et al., 2010; Gong et al., 2012). In the school environment, this conclusion has been confirmed by empirical research (Eun et al., 2012; Li et al., 2017). However, there is no relevant research on the relationship between preschool teachers’ proactive personality and creative behavior. In conclusion, employees with proactive personality will take active actions to change the surrounding environment in organizational work and may be more likely to exhibit innovative behaviors. Based on this, the following hypothesis is proposed in this study:

*Hypothesis 1*: Proactive personality is positively related to employee innovation behavior.

### Proactive Personality, Error Management Climate, and Innovative Behavior

In an organization, errors may occur from time to time. Practice has proved that because people have limited access to information when making decisions, mistakes cannot be completely prevented (Zhao and Olivera, 2006). Therefore, people begin to treat mistakes more scientifically (Van Dyck et al., 2005). An error management climate is an integral part of a positive organizational culture. Errors are defined as behaviors that unexpectedly deviate from goals or expectations. It is believed that the error management climate (EMC) is an organizational climate that can promote employees to communicate and share errors, help each other in the error environment, explore and analyze errors, reduce the negative impact of errors, and quickly reply from mistakes when mistakes occur (Cigularov et al., 2010).

Innovation refers to the process of an individual producing an entity and applying it to the organization, while creativity refers to the novel and meaningful ideas and perspectives that an individual generates in a particular field (Ford, 1996). Innovation occurs when creative ideas are successfully executed in an organization, so individual creativity is the basis of innovation (Amabile et al., 1996).

According to the creativity component theory, the generation of individual creativity not only requires relevant skills, motivation, etc., but also is affected by the external environment (Amabile, 1993). Therefore, as an important external environmental factor, the error management climate will inevitably have an impact on individual creativity and innovative behavior. When the organization has a high error management climate, it will create a more relaxed autonomy support environment. When employees are in this environment, they will have a stronger sense of responsibility and higher intrinsic motivation (Oldham and Richard Hackman, 2010). The research of Oldham and Cummings (1996) also believes that, compared with the high controlling organizational climate, more autonomous and supportive work has a significant positive impact on the innovative performance of employees by improving the level of motivation.

In the context of kindergarten, organizational climate refers to teachers’ perception of the overall environment and ideology of the school (Thomas, 1976; Dutta and Sahney, 2016). Teachers directly or indirectly perceive events, activities, and procedures in the workplace (Smith et al., 1969). When such cognition becomes a form of common cognition among kindergarten teachers, it becomes part of the organizational atmosphere of the kindergarten. As leaders of kindergartens, decision makers of organizations and creators of organizational culture, principles of kindergartens play an important role in the formation of organizational climate, and the establishment of a positive environment (Anggraini et al., 2018). In a school working environment, the school climate is a relatively enduring quality throughout the school, which describes the participants’ collective perception of daily behavior and influences their attitudes and behavior in school (Townley, 1991). Research shows that creating a positive, open climate has many benefits, including improved student achievement (Hoy et al., 1998), and ratings of school effectiveness (Townley, 1991).

Seibert and Kraimer (2001) believe that employees with proactive personality tend to have more innovative thinking and innovation ability at work. Research has found that employee innovation behavior is also closely related to individual psychological characteristics. For example, some studies have also shown that individual positive emotional factors (Conway and Engle, 1994; Ashby et al., 1999; Fredrickson, 2001), job dissatisfaction attitude (George and Zhou, 2001), self-efficacy (Mathisen and Bronnick, 2009), and other factors are significantly correlated with employee innovation behavior.

The innovative behavior of employees is not only related to individual characteristics but also closely related to the environment. Córdoba-Vega and Naranjo-Valencia (2017) through empirical research, it is found that employee innovation behavior is significantly affected by organizational culture. Jaskyte and Kisieliene (2006) found that among knowledge workers, there is a significant positive correlation between employee innovation behavior and organizational culture; that is, the stronger the organizational learning climate is the greater the impact on innovative behavior.

Thus, the specific hypotheses tested in this study include the following:

*Hypothesis 2*: Error management climate mediates the relationship between proactive personality and employee innovation behavior.

*Hypothesis 2a*: Proactive personality is positively related to error management climate.

*Hypothesis 2b*: Error management climate is positively related to employee innovation behavior.

### Proactive Personality, Self-Efficacy, and Innovative Behavior

Bandura (1989) first proposed “self-efficacy” and defined it as an individual belief that he or she can accomplish a certain task. Self-efficacy, self-esteem, and optimism are considered to be three components of personal resources that contribute to positive human behavior (Demerouti et al., 2001; Bakker and Demerouti, 2007; Xanthopoulou et al., 2007).

Research has confirmed that people with high levels of self-efficacy may be able to handle difficult tasks and also tend to get valuable results through persistence, which in turn generates intrinsic satisfaction from their work (Borgogni et al., 2013; Peng and Mao, 2015). In the school environment, some studies have found that self-efficacy is related to innovative behavior (Nafees et al., 2019; Soni and Bakhru, 2019). In kindergarten, teachers’ self-efficacy can produce more innovative behaviors and improve teaching quality. For example, in music education, preschool teachers use different teaching aids creatively to realize children’s perception of music (Jan and Shyan, 2010; Lenzo, 2014).

In this study, we focus on the relationship between self-efficacy and innovative behavior of preschool teachers. Few current studies directly focus on the relationship between proactive personality and self-efficacy. Xanthopoulou et al. (2009) believe that self-efficacy is different from other personality traits; it is malleable and can change with the change of environment.

According to the theory of social cognition and the model of three-way interaction, the subject, behavior and environment have dynamic interaction and influence each other. Under the influence of the environment, the role of individual factors on behavior is more prominent (Bandura et al., 1999).

Griffin et al. (2007) believe that people with proactive personality are self-initiated, change oriented, and future-oriented. They are relatively more innovative and likely to elicit greater feelings of self-confidence and self-efficacy (Hsieh and Huang, 2014). The research of Fay and Frese (2001) also confirmed this hypothesis. They found that proactive personalities can promote individuals feelings of self-efficacy, which in turn motivates their behavior and outcomes.

Tierney and Farmer (2002) believed that people with a high sense of self-efficacy, especially those with a high sense of self-efficacy in innovation, would get a higher innovation result. Ford (1996) constructed a conceptual model of individual creative behavior, taking efficiency information as a key motivational factor. Taylor and Popma (1990) found that efficacy beliefs have a positive effect on academic creative behavior of university professors. The research results on the relationship between innovative self-efficacy and innovative behavior also show that self-efficacy has a positive effect on individual innovative behavior.

Therefore, the specific hypotheses tested in this study include the following:

*Hypothesis 3*: Self-efficacy mediates the relationship between proactive personality and employee innovation behavior.

*Hypothesis 3a*: Proactive personality is positively related to self-efficacy.

*Hypothesis 3b*: Self-efficacy is positively related to employee innovation behavior.

### Error Management Climate and Self-Efficacy

Errors are inevitable, so the negative consequences should be reduced and the positive effects of errors should be magnified as the core idea of the error management atmosphere (Van Dyck et al., 2005). Any innovative activities and behaviors are the results of behaviors under the uncertain environment (Frese and Keith, 2015). The basic premise in the system approach is that humans are fallible and errors are to be expected, even in the best organizations (Reason, 2000). In a school environment, leaders, teachers, and students make mistakes in many ways. In kindergarten, preschool teachers inevitably make mistakes when they carry out innovative activities.

According to social information processing theory, individual psychology and behavior are not only determined by individual needs or goals but also influenced by environmental factors (Salancik and Pfeffer, 1978). Gurbin (2015) also believes that the individual information processing process is to a large extent affected by the social and cultural environment where the individual is, such as the organization, national culture, personal preference, and other factors; this influence runs through the whole process of information processing. According to social information processing theory, individual self-perception is mainly influenced by individual attribution style.

The organizational climate perceived by employees will provide an important basis for the attitude and behavior of employees. In kindergartens, when preschool teachers make mistakes, they can feel the organizational atmosphere of tolerance, so the creative behaviors of teachers will be further encouraged. According to the stimulus-cognition-response model (S-C-R) proposed by Tolman, cognition is considered to be an individual process of organizing and interpreting information from the outside world (Young and Tolman, 1933). Employees’ creativity and self-efficacy are easily affected by the work environment and atmosphere. As an organizational environment, the error management atmosphere will undoubtedly affect employees’ self-efficacy and innovative behavior (Eder and Eisenberger, 2008). When the organizational environment is shown as support and encouragement, the external pressure of individuals will be released, and the motivation of individual innovation behavior will be improved.

In conclusion, we put forward the following hypotheses:

*Hypothesis 4*: Error management climate is positively related to self-efficacy.

*Hypothesis 5*: Error management climate and self-efficacy sequentially mediate the relationship between proactive personality and employee innovation behavior.

**Figure 1:**
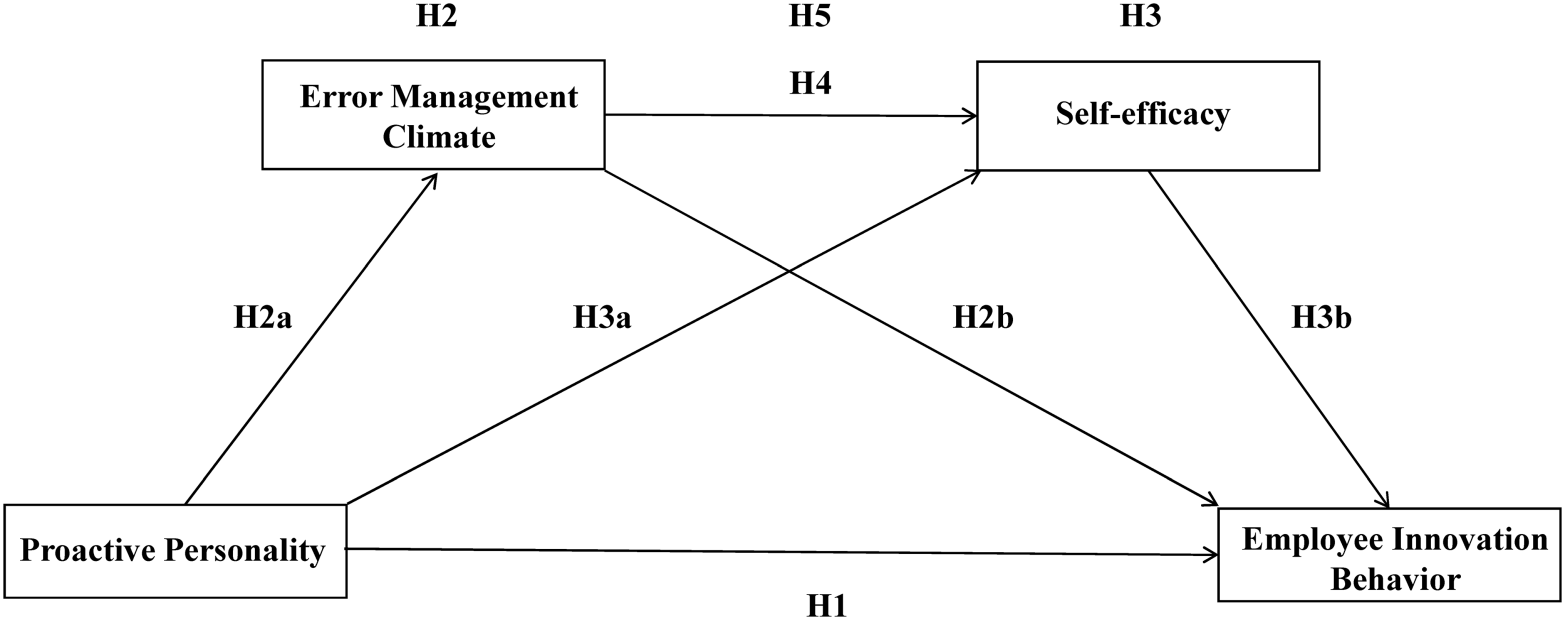
Theoretical hypotheses.

A conceptual model of error management climate and self-efficacy sequentially mediate the relationship between proactive personality and employee innovation behavior is presented in Figure 1. In addition, the conceptual model presents also the hypotheses under study.

## Materials and Methods

### Participants

This study adopts convenient stratified sampling, sampling kindergartens of different natures in five districts of Jinan City, Shandong Province. The kindergarten teachers participating in this survey are all full-time teachers on duty. To ensure the representativeness of the sample, the demographic distribution characteristics of the kindergarten teachers participating in the survey are analyzed. The formal test will be conducted by preschool education interns who have received unified training. After communicating with the kindergarten’s leader, the questionnaire will be distributed and collected online. Unified instructions were used for each test, and the confidentiality of the survey was emphasized to ensure the validity and authenticity of the questionnaire. A total of 500 questionnaires were sent out, of which 458 were actually recovered. After eliminating the serious data missing and incomplete questionnaires, 439 were effectively received with an effective recovery of 87.8%. Among them, 14 preschool teachers (3.2%) were male, 425 (96.8%) were female, 141 (32.1%) were public kindergartens, and 298 (67.9%) were private kindergartens. In terms of the level of the park, 102 people (23.2%) were in the provincial first-level park, 167 people (38%) were in the provincial second-level park, and 170 people (38.7%) were in the provincial third-level park. In terms of teaching age, the average teaching age was 3.506years and the SD was 1.477. Before analysis, the kurtosis and skewness of the data were tested, the main scale distribution shows enough normality indices; this allows us to take this into consideration for more in-depth statistical inferential analyses.

### Measures

#### Proactive Personality Scale

Yang and Chau (2016) revised the Proactive Personality Scale. The revised Proactive Personality Scale contains 10 items. The Chinese version was revised by Shang and Gan (2009). The results also show that the Chinese version of the scale has good convergent validity, discriminant validity, and predictive validity. A sample item was “Wherever I have been, I have been a powerful force for constructive change.” The survey questionnaire was measured with a seven-scale Likert scale. The questionnaire was measured with seven-point Likert scale (1=strongly inconsistent and 7=strongly consistent). In this study, Cronbach’s *α* value was 0.917.

#### Error Management Climate Scale

Van Dyck et al. (2005) divided the error management climate into four dimensions, namely error learning (five items), error thinking (five items), error ability (five items), and error communication (five items), with a total of 20 items. The scale has been proved to have good reliability and validity in China (Chen et al., 2020). The sample items were “If team members are unable to continue their work after an error, they can rely on others” and “When mastering a task, team members can learn a lot from their mistakes.” Survey questionnaire was measured with seven-point Likert scale for measuring (1=very does not comply and 7=very accord with), the higher the score indicates that employees within the organization on error management climate, the higher the perception. In this study, Cronbach’s *α* value was 0.851, and the Cronbach’s *α* value of the four dimensions were 0.715, 0.716, 0.722, and 0.727, respectively.

#### General Self-Efficacy Scale

Measurement of Self-efficacy used the General Self-efficacy Scale (GSES), which was originally compiled by Schwarzer and Jerusalem (1995). The scale has been proved to have good reliability and validity in China (Zhang and Schwarzer, 1995). A sample item was “It is easy for me to stick to my aims and accomplish my goals.” The scale had 10 questions and adopted Likert four-point scoring method (1=completely incorrect and 4=completely correct). In this study, Cronbach’s *α* value was 0.806.

#### Scale of Employee Innovative Behavior

The six-item Scale of employee innovative behavior developed by Scott and Bruce (1994). This scale has good reliability and validity in China and is used in many empirical studies (Cao and Zhang, 2020; Hou et al., 2020). A sample item was “Promotes and champions ideas to others.” The survey questionnaire was measured with a seven-scale Likert scale, and the respondents were asked to evaluate their own innovation performance. The questionnaire was measured with seven-point Likert scale (1=strongly inconsistent and 7=strongly consistent), with higher scores indicating higher creative performance. In this study, Cronbach’s *α* value was 0.796.

### Statistical Methods and Analysis Ideas

In this study, SPSS22.0 and MplusVersion 8.3 were used for data analysis, among which SPSS was mainly used for data consolidation and descriptive statistical analysis. Mplus is mainly used for model verification. Participants missing descriptive data or missing many data points were dealt with when running the analysis by means of list wise deletion. We take the gender of preschool teachers, teaching years, kindergarten nature, and kindergarten grade as the control variables. Gender was dummy coded (0=female and 1=male), the nature of kindergarten was dummy coded (0=public kindergarten and 1=private kindergarten).

## Results

### Test of Common Method Deviation

Using Harman single factor test method, 10 factors with characteristic root greater than 1 were obtained. The explanation rate of the first factor is 22.10%, which is less than the cut-off value of 40% (Podsakoff et al., 2003), indicating that there is no significant common method bias in this study.

### Descriptive Statistical Analysis

Table 1 lists the major variables and Pearson correlation coefficients between each dimension. As can be seen from Table 1, employee innovation behavior is significantly positively correlated with proactive personality, error management atmosphere, and self-efficacy. According to the views of Tsui et al. (1995), the critical value of the correlation level with serious multicollinearity problems is generally more than 0.75. In this study, the correlation coefficient of all variables is less than 0.6, and there is no serious multicollinearity problem among the major variables.

**Table 1:** Means, SDs, and correlations of the major study variables.

Variable	M	SD	1	2	3	4	5	6	7	8
1. Gender	0.030	0.176	1							
2. Teaching age	3.510	1.477	0.017	1						
3. Kindergarten nature	0.680	0.467	−0.153^**^	−0.300^**^	1					
4. Kindergarten grade	2.150	0.773	0.081	0.163^**^	−0.146^**^	1				
5. PP	3.748	1.314	0.034	−0.017	0.07	−0.252^**^	1			
6. EMC	4.320	0.775	0.013	−0.046	0.066	−0.034	0.341^**^	1		
7. SE	2.560	0.624	0.021	−0.035	0.176^**^	−0.233^**^	0.300^**^	0.304^**^	1	
8. EIB	4.530	0.959	0.048	−0.076	0.104^*^	−0.189^**^	0.507^**^	0.590^**^	0.455^**^	1

** *p<0.01*;

* *p<0.05*.

*N=439. PP, proactive personality; EMC, error management climate; SE, self-efficacy; EIB, employee innovation behavior. Gender is the dummy variable (0=female and 1=male); The kindergarten nature is a dummy variable (0=public kindergarten and 1=private kindergarten)*.

### Model Inspection

The model was fitted by Mplus, the fitting index of the model was ML *χ*^2^=954.141, *df* = 507, *χ*^2^/*df* = 1.882, CFI=0.914, TFI=0.906, RMSEA=0.045, SRMR=0.054. Each index is in an acceptable range, and the model is ideal. See Table 2.

**Table 2:** Fit indices of the model.

Fit indices	Recommended threshold	Scores	Remarks
ML *χ*^2^	-	954.141	-
Df	-	507	-
*χ*^2^/*df*	1< *χ*^2^/*df* <3	1.882	Acceptable
CFI	>0.9	0.914	Acceptable
TLI	>0.9	0.906	Acceptable
RMSEA	<0.08	0.045	Acceptable
SRMR	<0.08	0.054	Acceptable

### The Significance Test of Mediating Effect

On the basis of good model fitting, the Bootstrap program of Mplus was used to repeat the sample for 5,000 times. The results show that the path coefficients of proactive personality, error management climate, self-efficacy, and employee innovation behavior are all significant.

Proactive personality is positively related to employee innovation behavior (*β*=0.309, *p*<0.001), supporting H1. Proactive personality is positively related to error management climate (*β*=0.374, *p*<0.001), supporting H2. Error management climate is positively related to employee innovation behavior (*β*=0.44, *p*<0.001), supporting H2b. Proactive personality is positively related to self-efficacy (*β*=0.198, *p*=0.002), supporting H3a. Self-efficacy is positively related to employee innovation behavior (*β*=0.318, *p*<0.001), supporting H3b. Error management climate is positively related to self-efficacy (*β*=0.248, *p*<0.001), supporting H4. See Table 3.

**Table 3:** The direct effect of the research paths and research model hypothesis analysis.

DV	IV	Std. Est.	S.E.	Est./S.E.	*p*	*R* ^2^	Hypo and path	Remarks
EIB	PP	0.309	0.045	6.859	***	0.644	H1:PP→EIB	Support
	EMC	0.44	0.051	8.617	***		H2b:EMC→EIB	Support
	SE	0.318	0.058	5.477	***		H3b:SE→EIB	Support
EMC	PP	0.374	0.054	6.881	***	0.151	H2a:PP→EMC	Support
SE	PP	0.198	0.062	3.162	0.002	0.201	H3a:PP→SE	Support
	EMC	0.248	0.062	3.977	***		H4:EMC→SE	Support

*** *p<0.001*.

Table 4 shows the indirect effects of the study path. Error management climate mediates the relationship between proactive personality and employee innovation behavior (*β*=0.104, *p*<0.001), with 95% CI (0.066–0.156), excluding 0, supporting H2, and the mediating effect accounted for 29.05%.

**Table 4:** The indirect effect of the research paths.

Path	Std. Est.	S.E.	Est./S.E.	*p*	Boot LLCI	Boot ULCI	The proportion of the effect (%)
H2:PP→EMC→EIB	0.104	0.022	4.638	***	0.066	0.156	29.05
H3:PP→SE→EIB	0.04	0.015	2.686	0.007	0.015	0.074	11.17
H5:PP→EMC→SE→EIB	0.019	0.007	2.851	0.004	0.009	0.035	5.31
TOTALIND	0.162	0.026	6.313	***	0.117	0.219	45.25
TOTAL	0.358	0.042	8.513	***	0.281	0.447	100.00

*** *p<0.001*.

Self-efficacy mediates the relationship between proactive personality and employee innovation behavior (*β*=0.04, *p*=0.007), with 95% CI (0.015–0.074), excluding 0, supporting H3, and the mediating effect accounted for 11.17%.

**Figure 2:**
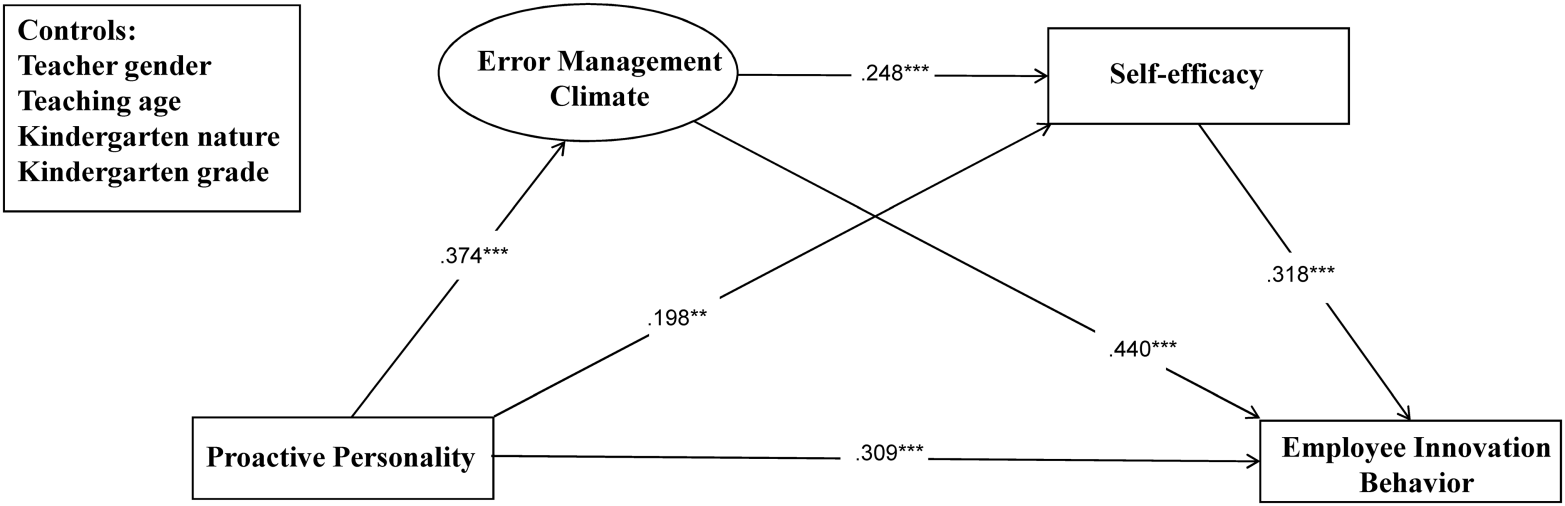
Structural equation.

Error management climate and self-efficacy sequentially mediate the relationship between proactive personality and employee innovation behavior (*β*=0.019, *p*=0.004), with 95% CI (0.009–0.035), excluding 0, supporting H5, and the mediating effect accounted for 5.31%. See Figure 2.

## Discussion

From the aspect of proactive personality trait and organizational level, this study probes into preschool teachers’ proactive personality and innovative behavior in kindergarten and its influencing mechanism. Previous studies have found that proactive personality is related to employees’ innovative behavior (Giebels et al., 2016; Fumeng, 2018; Afsar et al., 2020) and self-efficacy (Travis and Freeman, 2017; Premchandran and Priyadarshi, 2019). Error management atmosphere is also related to employee innovation behavior (Hagen, 2018). However, no relevant studies have investigated the sequential indirect effects of error management atmosphere and self-efficacy on proactive personality and employee innovation behavior. This study found that the proactive personality does not predict individual innovation behavior by itself, but indirectly influences it, through the mediating effect of the climate of error management and self-efficacy. This is an important contribution to the research on the relationship between proactive personality and innovative behavior.

In addition, another important finding of this study is that error management is the factor that plays a decisive role in creating a climate of innovation, much more than self-efficacy. This research result has important theoretical contribution. According to the theory of creativity composition, the generation of individual creativity not only requires relevant skills and motivations but also is affected by external environment (Amabile, 1993). However, no relevant research has proved that individual internal motivation, especially individual self-efficacy and external environment which factor plays a higher degree of influence or decisive role. This finding will provide better empirical evidence for innovation theory.

Proactive personality plays an important role in promoting employee innovation behaviors. In kindergartens, preschool teachers with proactive personality should give full play to their important role in curriculum innovation, teaching activity innovation, and kindergarten organization innovation. Only by fully mobilizing their innovation abilities and innovation level we can further improve the innovation ability of kindergartens. The mediating effect shows that proactive personality can not only directly predict individual innovation behavior but also indirectly influence individual innovation behavior through the chain mediating effect of error management climate and self-efficacy. Organizations with a good error management atmosphere view errors as inevitable, accept this reality and optimize their workflow based on it. Employees’ creativity is often affected by the surrounding environment (Sokol et al., 2015).

An environment that provides a framework for innovative action, consistent with the core characteristics associated with creative activity, seems likely to facilitate innovation. Thus, supporting autonomy or building organizations and educational systems that tolerate error may increase the likelihood of innovative achievement. However, given the climate, some different emergency measures may be required, depending on the style of innovation one wants to encourage. An atmosphere that encourages risk-taking and open questioning, while emphasizing the value of different experiences, multiple understandings, new understandings, and even a total acceptance and tolerance of wrongdoing, seems more useful. Good kindergartens should pay special attention to organizational environment for innovation behavior to promote preschool teachers and the formation of good atmosphere of error management, error of preschool teachers’ behavior is a tolerant attitude, further high employee self-efficacy, advocacy of preschool teachers learn by mistakes nothing wrong, wrong thinking, communication, further development of innovative behavior. Preschool teachers are not only the main body of individual innovation, the main body of curriculum innovation and the formation of the core competitiveness of kindergartens, but also the important activity leader and important influence in the education link, whose innovation ability and innovation level determine the level of our early childhood education, determine the quality of early childhood education. The formation of kindergarten innovation atmosphere and good kindergarten mental health environment need leaders to be tolerant of individual mistakes, need to have a sound error management mechanism and good error management atmosphere.

## Limitations and Future Research Directions

Firstly, the results are from self-reported data, and future studies might consider using more objective indicators. Secondly, the methods used in this study are horizontal and do not reflect the long-term performance of the mechanisms examined in this study. Particularly, in an emergency context due to the COVID-19 pandemic which is having a relevant impact on the experience of work, underlining how urgent it is to promote workers’ proactive role in error management at work (Galanti, 2021). Future research should take into account the error management of kindergarten organizations in emergency situations caused by the COVID-19 pandemic. Finally, the study comprises possible mediators but other mediating effects from other variables should be included in the relationship between both constructs. In the unique environment of kindergartens, future research should also consider the importance of individual and organizational contributions to organizational safety management, emphasize the existing links between safety promotion and employee motivation and involvement (Galanti et al., 2021) and examine the impact of organizational error management climate on preschool teachers.

## Conclusion

The findings of this study suggest that proactive personality is significantly and positively related to innovative behavior. Proactive personality not only affects innovation behavior through error management climate but also affects innovation behavior through self-efficacy. In addition, the most important finding of this study is that error management climate and self-efficacy play a chain intermediary role in the relationship between preschool teachers’ proactive personality and innovative behavior. Moreover, among the two sequential mediating factors, error management is the factor that plays a decisive role in creating a climate of innovation, much more than self-efficacy. We believe that these findings can contribute to our research the literature on innovative behavior as well as management practices.

## Data Availability Statement

The raw data supporting the conclusions of this article will be made available by the authors, without undue reservation.

## Ethics Statement

The studies involving human participants were reviewed and approved by the Research Ethics Committee of the Wenzhou University. Written informed consent to participate in this study was provided by the participants. The patients/participants provided their written informed consent to participate in this study.

## Author Contributions

BP designed, prepared, carried out the data collection process, and written the article. ZS revised the section of the analysis and discussion and corrected the entire manuscript. YW analyzed and verified the data in this article. All authors contributed to the article and approved the submitted version.

## Funding

This work was supported by the second batch of teaching reform research projects of higher education in Zhejiang Province during the “13th Five-Year Plan” (project number: jg20190401). This project is supported by the Graduate Research and Innovation Fund of Wenzhou University.

## Conflict of Interest

The authors declare that the research was conducted in the absence of any commercial or financial relationships that could be construed as a potential conflict of interest.

## Publisher’s Note

All claims expressed in this article are solely those of the authors and do not necessarily represent those of their affiliated organizations, or those of the publisher, the editors and the reviewers. Any product that may be evaluated in this article, or claim that may be made by its manufacturer, is not guaranteed or endorsed by the publisher.
